# Outcomes of Dialysis Among Patients With End-Stage Renal Disease (ESRD)

**DOI:** 10.7759/cureus.17006

**Published:** 2021-08-08

**Authors:** Ayesha Ejaz, Abdul Manan Junejo, Muhammad Ali, Ahsan Ashfaq, Abdul Rauf Hafeez, Sadaqat Ali Khan

**Affiliations:** 1 Nephrology, Jinnah Postgraduate Medical Centre, Karachi, PAK; 2 Nephrology, Fazaia Ruth Pfau Medical College, Karachi, PAK; 3 Physiology, Liaquat National Medical College, Pakistan., Karachi, PAK; 4 Nephrology, Sindh Institute of Urology and Transplantation, Karachi, PAK; 5 Urology, Behram Medical Center, Kohat, PAK

**Keywords:** dialysis, end-stage renal disease, outcomes, mortality, survival

## Abstract

Background

Dialysis-associated morbidity and mortality among end-stage renal disease (ESRD) patients has been increasing, despite the advancement in pharmacological treatment and dialysis technology. The aim of this study was to determine the outcomes of dialysis among ESRD patients presenting at the nephrology department of Jinnah Postgraduate Medical Centre (JPMC).

Methodology

This cross-sectional study was conducted during the year 2015-2016, including 105 ESRD patients. Data were collected through a structured questionnaire inquiring about patient's demographics and hemodialysis details. The outcomes in terms of survival and death within one month of dialysis were also recorded. The statistical analysis was carried out using SPSS version 21.0 (IBM Corp, Armonk, NY).

Results

Gender distribution showed that most of the study patients were males (58.1%). The mean duration of ESRD was 7.65 ± 3.69 months while the mean duration of hemodialysis was 36.5 ± 5.65 hours. Among the comorbid conditions, hypertension (69.5%) and diabetes (64.8%) were the most prevalent, followed by renal stones, chronic pyelonephritis, and chronic nephritis. The outcomes indicated mortality among 16.2% of patients; all deceased ESRD patients had diabetes (p < 0.05). Moreover, the duration of hemodialysis was significantly associated with the outcomes of dialysis (p < 0.05).

Conclusion

In conclusion, a considerable mortality rate was observed among ESRD patients undergoing hemodialysis. Moreover, patient survival was better with the increased duration of dialysis.

## Introduction

Chronic kidney disease (CKD) is an enormously affecting public health condition characterized by structural abnormalities, impaired renal function, and persistent urine-associated abnormalities [1]. The sufferers are mostly at risk of obtaining associated systemic complications and, more severely, death may also occur [1]. Progressive CKD causes irreversible damage to kidney function, leading to ESRD, which affects the overall mortality and morbidity rate, health-related quality of life, requirement of health services, and treatment cost [2].

CKD affected 697.5 million people (9.1%) in 2017, with a relatively increased number of cases progressing towards ESRD [3]. With the accelerating frequency, the treatment cost also escalated based on advancement, availability, and accessibility [4]. Renal replacement therapy (RRT) is considered a life-saving treatment for ESRD patients, either through dialysis or kidney transplant [5]. But due to a lack of renal replacement services, only 2.5 million patients receive RRT while the majority remain unattended, i.e. 2.3-7.1-million adults expired prematurely due to lack of treatment accessibility [4]. Due to the high ESRD burden and financial instability among low and middle-income countries, access to RRT is much lower as compared to that in developed countries, i.e. over 80% of the patients receiving RRT belong to high-income countries [6].

There are diverse etiological factors associated with ESRD that may vary individually. Among the major risk factors are increasing age, comorbidities, including diabetes and hypertension [7]. Moreover, the overuse of certain analgesics for a longer period also causes analgesic nephropathy and damages the kidneys. Stones and cancers might also impact kidney function by urinary tract obstruction; obesity is also a rare cause [8]. Among all, diabetes stands as the largest single cause of ESRD, accounting for almost 30%-40% of all cases [9]. Evidently, ESRD-associated morbidity and mortality are comparatively more significant among the elderly than younger individuals receiving RRT. Together with age, delayed consultation and treatment initiation lead to increased risk [10]. It was found that 59% of dialysis patients aging ≥ 75 years of age die within a year and 43% within two years in the United States of America (USA) [10]. Whereas in developing countries like Pakistan, the mortality rate is comparatively higher due to limited resources and failure to fulfill treatment needs [11].

As mentioned, the pre-dialysis care and timing of dialysis initiation play an essential role in managing ESRD patients [12]. Late referral (LR), i.e. less than one to six months interval between the first consultation and RRT initiation, is associated with a high prevalence of uremic complications and socioeconomic cost [13]. On the contrary, early referral of patients to a nephrologist, i.e. more than a year before RRT begins, is associated with improved survival, improved quality of life (QoL), and lower medical costs [12-13]. The late referral is a substantial problem associated with ESRD management with no improvements, specifically in Pakistan. In case of inappropriate evaluation and delayed treatment, early referral to a nephrologist is highly recommended.

Moreover, under more severe circumstances, regular follow-up by a nephrologist is suggested, i.e. for patients with CKD Stages 4 and 5. The outcomes of dialysis in such patients within one month have not been studied in the local population. Moreover, dialysis withdrawal is the second or third leading cause of death among ESRD patients on RRT [14], accounting for 15%-22% of the deaths [10].

The study objective was to estimate the magnitude of mortality among ESRD patients with late referrals so that strategies could be designed to institute early preventive measures and improve medical care among such patients by reducing delayed referrals that will ultimately decrease the associated mortality and socioeconomic burden.

## Materials and methods

This cross-sectional study was conducted at the nephrology department of Jinnah Postgraduate Medical Centre (JPMC), Karachi, Pakistan, for a year from 2015-2016. The sample size of 105 was calculated using the STEPS Sample Size Calculator [15] based on the prevalence of mortality in late referrals undergoing hemodialysis [16]; by keeping a confidence level of 95% and 7% margin of error.

All ESRD patients between 15 and 60 years of age presenting for the first time to the nephrology unit of JPMC with advanced uremia and academia were included in the study. In contrast, patients who were already on hemodialysis and those with acute or chronic renal failure were kept in the exclusion criteria. Ethical approval was obtained from the institutional ethical review committee and written informed consent was taken from each patient before study initiation. Each patient underwent hemodialysis under the supervision of a trained physician. Data were collected using a structured questionnaire inquiring about the demographic and hemodialysis details. The hemodialysis frequency and duration of each patient were noted. Outcomes in terms of survival and death within one month of dialysis were also recorded.

Data were statistically analyzed using SPSS version 11.0 (SPSS Inc., Chicago, IL); the mean and standard deviation were calculated for all continuous variables, including age, duration of ESRD, and hemodialysis, while categorical variables like gender, frequency of hemodialysis sessions per week, and outcomes were presented using frequency and percentages. A chi-square test was used to assess the association between the study variable. Data were stratified for age, gender, comorbidities (hypertension, diabetes, chronic nephritis, renal stones, and chronic pyelonephritis), duration, and frequency of hemodialysis sessions to see their effect on outcomes (survival or mortality). P-value <0.05 was considered statistically significant.

## Results

A total of 105 ESRD patients were included in the study; gender distribution indicates that most of the patients were males, i.e. 58.1%. The enrolled patients' mean age was 52.14 ± 6.65 years, with 70.5% of patients > 50 years of age. The mean duration of ESRD was found to be 7.65 ± 3.69 months, and the mean duration of hemodialysis was 36.5 ± 5.65 hours. Of the total, 56.19% of patients had a hemolysis duration of ≤ 35 hours, while 43.80% of patients had > 35 hours of hemodialysis duration. Regarding the frequency of hemodialysis sessions per week, 69.52% of patients had two dialysis sessions per week, while 30.47% had dialysis thrice per week. Among the outcomes, only 16.2% of patients died while 83.2% survived (Table 1).

**Table 1:** Baseline characteristics of the study population Values are given as mean ± SD or n (%)

Variables		(n=105)
Age (Years)		52.14±6.65
Mean duration of ESRD (Months)		7.65±3.69
Mean duration of Hemodialysis (Hours)		36.5±5.65
Age Groups	≤ 50 years	31(29.5)
	> 50 years	74(70.5)
Gender	Male	61(58.1)
	Female	44(41.9)
Duration of Hemodialysis	≤ 35 hours	59(56.19)
	>35 hours	46(43.80)
Frequency of Hemodialysis (per week)	Twice	73(69.52)
	Thrice	32(30.47)
Outcomes	Survived	88(83.2)
	Expired	17(16.2)

**Figure 1:**
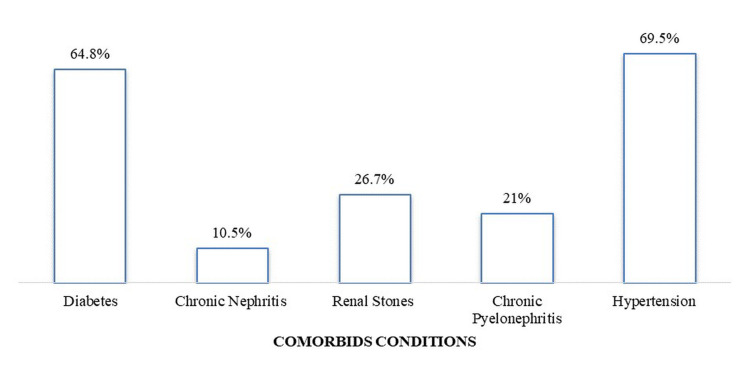
Common comorbidities among enrolled ESRD patients ESRD: end-stage renal disease

Figure 1 shows that the most common comorbid conditions among the studied subjects were hypertension (69.5%) and diabetes (64.8%), followed by renal stones (26.7%), chronic pyelonephritis (21%), and chronic nephritis (10.5%).

Among the comorbidities, diabetes and renal stones were significantly associated with the outcomes (p < 0.05). In contrast, either no or marginal association was found between the other comorbid conditions like chronic pyelonephritis, hypertension, and chronic nephritis and dialysis outcomes (Table 2).

**Table 2:** Association of comorbid condition with outcomes Values are given as n(%). *p-value<0.05 is considered significant.

Variables		Outcomes		p-value
		Survived (n=88)	Expired (n=17)	
Diabetes	Yes	51(57.90)	17(100)	0.001*
	No	37(42.04)	-	
Chronic Nephritis	Yes	6(6.81)	5(29.41)	0.160
	No	82(93.18)	12(70.58)	
Renal Stones	Yes	18(20.45)	10(58.82)	0.002*
	No	70(79.54)	7(41.17)	
Chronic Pyelonephritis	Yes	17(19.31)	5(29.41)	0.262
	No	71(80.68)	12(70.58)	
Hypertension	Yes	58(65.90)	15(88.23)	0.060
	No	30(34.09)	2(11.76)	

As per the stratification, 12.9% of patients with ≤ 50 years of age and 17.5% of patients with >50 years of age expired. Gender-wise distribution displayed that 19.7% of male patients and 11.4% of female patients expired while 55.68% of males and 44.31% of females survived. Moreover, the mortality rate was higher among patients with ≤ 35 hours of hemodialysis duration, indicating a significant effect of hemodialysis duration on the outcomes (p = 0.001). Mortality was marginally higher among the patients undergoing dialysis twice per week, i.e. 16.43% while 15.62% of patients having dialysis sessions thrice per week expired (Table 3).

**Table 3:** Factors influencing the outcomes Values are given as n (%). *p-value <0.05 is considered significant.

Variables		Outcomes		p-value
		Survived (n=88)	Expired (n=17)	
Age	≤ 50 years	27(30.68)	4(12.9)	0.554
	> 50 years	61(69.31)	13(17.5)	
Gender	Male	49(55.68)	12(19.7)	0.254
	Female	39(44.31)	5(11.4)	
Duration of Hemodialysis	≤ 35 hours	43(48.86)	16(27.1)	0.001*
	> 35 hours	45(51.13)	1(2.2)	
Frequency of Hemodialysis	Twice	61(69.31)	12(16.43)	0.191
	Thrice	27(30.68)	5(15.62)	

## Discussion

The significance of pre-dialytic care and duration of hemodialysis initiation in overcoming ESRD-associated mortality and morbidity is largely known and understood by nephrologists as compared to other health workers outside the nephrology community [17]. It was found that non-nephrologists were usually unsure regarding the indications and timings of the referral. The delayed referral and treatment duration frequently raise complications that may be gastrointestinal, cardiovascular, hematological, dermatological, and electrolyte imbalances leading to metabolic acidosis, etc. [18]. In their research, Kessler and colleagues compared the knowledge of nephrologists and other healthcare providers regarding referrals. They suggested that the cases with delayed referrals were observed to have low contact with primary care compared to those provided with early referral [19].

In our study, no significant gender-based differences influencing the outcomes, either mortality or survival among the enrolled ESRD patients were observed (Table 3). There were more male patients as compared to females in the present study; therefore, the differences in the mortality rate may be biased as per the dissimilar ratio, i.e. 19.7% males and 11.4% females died while 55.68% males and 44.31% females survived (p = 0.25). In contrast, a study indicated females as being at greater risk for death as compared to males [20].

Diabetes was the most reported comorbidity among enrolled patients (Figure 1). The literature also suggests that diabetes is the primarily reported comorbid condition among ESRD patients, and the two conditions seem to have a positive correlation [21]. A study in support revealed that out of 2006 diabetes patients, 38.3% suffered from diabetic nephropathy (DN) [21]. On the contrary, our findings display a higher frequency of diabetes among ESRD patients than the results published in a previous meta-analysis conducted by El Hafeez and colleagues [22]. Furthermore, in our study, all expired ESRD patients had a history of diabetes (Table 2). A study revealed a high mortality rate among octogenarians with poor functional status and comorbidities such as diabetes or hypertension, increasing the death risk up to 48% within a year [23]. In addition to diabetes, chronic nephritis, chronic pyelonephritis, and hypertension were the other less common comorbidities that had no significant effects on the dialysis outcomes (Table 2).

Another factor associated with high mortality is the duration of ESRD19; it has been reported that ESRD patients between 18 to 64 years of age expire within five years after the incidence [24]. The mean duration of ESRD in our study was found to be 7.65 ± 3.69 months, with mortality reported among 16.2% of the cases. Furthermore, the secondary factors that may affect the outcomes of RRT among ESRD patients include socioeconomic status, age, race. Kasiske et al., in their study, demonstrated that factors like race, ethnicity, and education are associated with the outcomes of treatment [25]. While in our study, none of these secondary factors showed a significant relationship with the outcomes of dialysis (Table 3).

There are some limitations to the current study that need to be addressed. We did not assess the negative effects of dialysis therapy among ESRD patients and the associated impact on the outcomes. Moreover, additional factors like other comorbid conditions apart from those listed in the study might have affected the enrolled patients' survival rate. Although the survival rate was high, it is recommended that large-scale studies involving the local population must be conducted for further confirmations.

## Conclusions

Our results indicated that mortality was observed among 16.2% of the ESRD patients on dialysis. A considerable mortality rate was observed among ESRD patients undergoing hemodialysis. Patient survival was better with the increased duration of dialysis. No gender-based differences were observed in the study outcomes while among the comorbid condition, diabetes was the most prominent. Moreover, there was a significant variation in the survival and death rate among diabetics and non-diabetics. Further long-term cohort studies and randomized control trials are required for better assessment and potential outcomes, also considering the QoL of the patient treated. A newer approach is required to provide better survival advantages and rehabilitation to ESRD patients on dialysis to improve the overall mortality rate.
